# Pattern of crop raiding by wild large mammals and the resultant impacts vary with distances from forests in Southwest Ethiopia

**DOI:** 10.1002/ece3.7268

**Published:** 2021-02-14

**Authors:** Alemayehu Mamo, Debissa Lemessa, Obsu Hirko Diriba, Debela Hunde

**Affiliations:** ^1^ College of Agriculture and Veterinary Medicine Jimma University Jimma Ethiopia; ^2^ Ethiopian Biodiversity Institute Addis Ababa Ethiopia

**Keywords:** biosphere reserve, crop raiding, Ethiopia, forest edge, perception, Yayu

## Abstract

Crop raiding is a major form of human‐wildlife interaction mainly in the ecotone areas of human‐modified natural landscapes. The aim of this study was to examine the spatial pattern of crop raiding and the resultant impacts on how farmers perceive forests at different distances from Yayu Coffee Forest Biosphere Reserve which is located in southwest Ethiopia. For this, thirty transects (each 1 km long) were laid out at 200 m interval parallel to forest edges: ten transects close to forest (<0.5 km), ten at intermediate (0.5–1 km), and ten transects were taken far from forest (>1 km). Along each transect, 2–6 households were randomly selected and interviewed using semistructured questionnaire. The perception of the respondents on forests at different distances from forest edges was analyzed using Pearson's Chi‐square test. The variation in the amount of damage among these three locations was tested using one‐way ANOVA. Four wild large mammals including olive baboon, vervet monkey, bush pigs, and crested porcupine were identified as top crop raiders in the area. The frequencies of occurrence of crop raiders decreased with increasing distance from forest edges. Similarly, the amount of damage in maize fields was higher close to forests when compared with that of either at intermediate or far from forest edges (*p* < .001). Eighty‐one percent of the households living close to the forests perceive that forest is a threat to their survival. Overall, our results imply that strategies need to be sought in order to minimize the socio‐ecological impacts of crop raiders mainly in locations close to forest edges.

## Graphical abstract

Crop raiding is decreasing with increasing distance from forest edges. The amount of damage in maize fields was higher close to forest edges Households living close to the forests perceive that forest is home for wild animals and a threat to their survival.

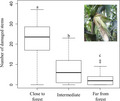

## 1 INTRODUCTION

Crop raiding‐literally a factor for a human‐wildlife conflict is largely a negative interaction that occurs when wild animals come out of their natural habitats (forests) to the crop fields or homegardens to rob the crops that the farmers have grown for their own and their families consumption (Hill, 2017; Madden, 2004). Such phenomenon has been in existence since humans and wild animals have begun sharing the same landscapes and resources. Nowadays, human‐wildlife conflict exists in one form or another (crop raiding, livestock depredation, property damage, and human injury) across the globe (Hariohay & Røskaft, 2015; Laverdière et al., 2007). In protected areas, the human‐wildlife conflict is severe and has become a growing challenge mainly due to the mismatches between the interests of conservation and local residents' livelihood improvement (Ango et al., 2017; Sillero‐Zubiri & Switzer, 2001). Hence, to minimize such mismatch, it needs to understand the socio‐ecological system pertinent to the human‐wildlife interaction mainly in biodiversity hotspot ecosystems (Hill, 2000).

In managing the human‐wildlife conflict, the attitude of the local inhabitants needs to be critically understood within temporal and spatial context, for instance, in areas where humans live adjacent to natural habitats and interaction often exists between humans and wild animals (Megaze et al., 2017). Crop raiding is a major cause of human‐wildlife conflict which thus influences the attitude of local people toward conservation of biodiversity (Hill, 2004). However, little is understood regarding the spatial pattern and the resultant socio‐ecological impacts of crop raiding by large wild mammals surrounding biodiversity hotspot areas of Ethiopia, for example, in biosphere reserve areas. Hence, we aimed to understand the pattern and the impacts of crop raiding at different distances from forest edges surrounding Yayu Coffee Forest Biosphere Reserve in southwest Ethiopia. Here, we hypothesized that the pattern of crop raiding frequencies of large wild mammals and the corresponding level of crop damage decrease with distance gradient from the edges.

Wild animals including insect pests, small and large mammals, and birds were reported to raid crops in Ethiopia (Quirin, 2005). On the other hand, the livelihood of local communities around protected areas mainly depends on agriculture which is very vulnerable to crop raiders. For example, Asmamaw and Verma (2013) reported that communities in and around Bale Mountain National Park heavily depend on livestock rearing and production of cereal and oil crops such as barley and linseed, vegetables and root crops including cabbages, potato, and carrots. Moreover, in southwest Ethiopia, several large mammals such as olive baboons, bush pigs, giant forest hogs, vervet monkeys, porcupines, warthogs, colobus, and blue monkeys were reported as a worst crop raiders in both field crop and homegardens (Lemessa et al., 2013; Ango et al., 2014). However, the pattern of the frequency of occurrence or raiding and the corresponding crop damage may vary with distance gradient from natural habitats into human‐modified landscapes (Lemessa et al., 2013; Madden, 2008). Moreover, the intensity of crop raiding depends on type of crop raider species, crop species grown, and season (Mwamidi et al., 2012). The study was executed by taking thirty transects (each 1 km long) which were laid out with 200 m interval from forest edge toward agricultural landscape. Moreover, these transects were categorized into three locations: ten transects close to forest (<0.5 km), ten intermediate (0.5–1 km), and ten transects far from forest (>1 km). Accordingly, from each transect, 2–6 households (i.e., 48 close to forest, 38 intermediate, and 38 far from forest households) and in total 124 households were randomly selected to undertake a household questionnaire using semistructured questionnaire. Altogether, the results showed that the pattern of crop raiding, the extent crop damage and how farmers perceive forests vary with distances from the forest edge surrounding Yayu Coffee Forest Biosphere reserves.

## 2 MATERIALS AND METHODS

### 2.1 Study area

**FIGURE 1:**
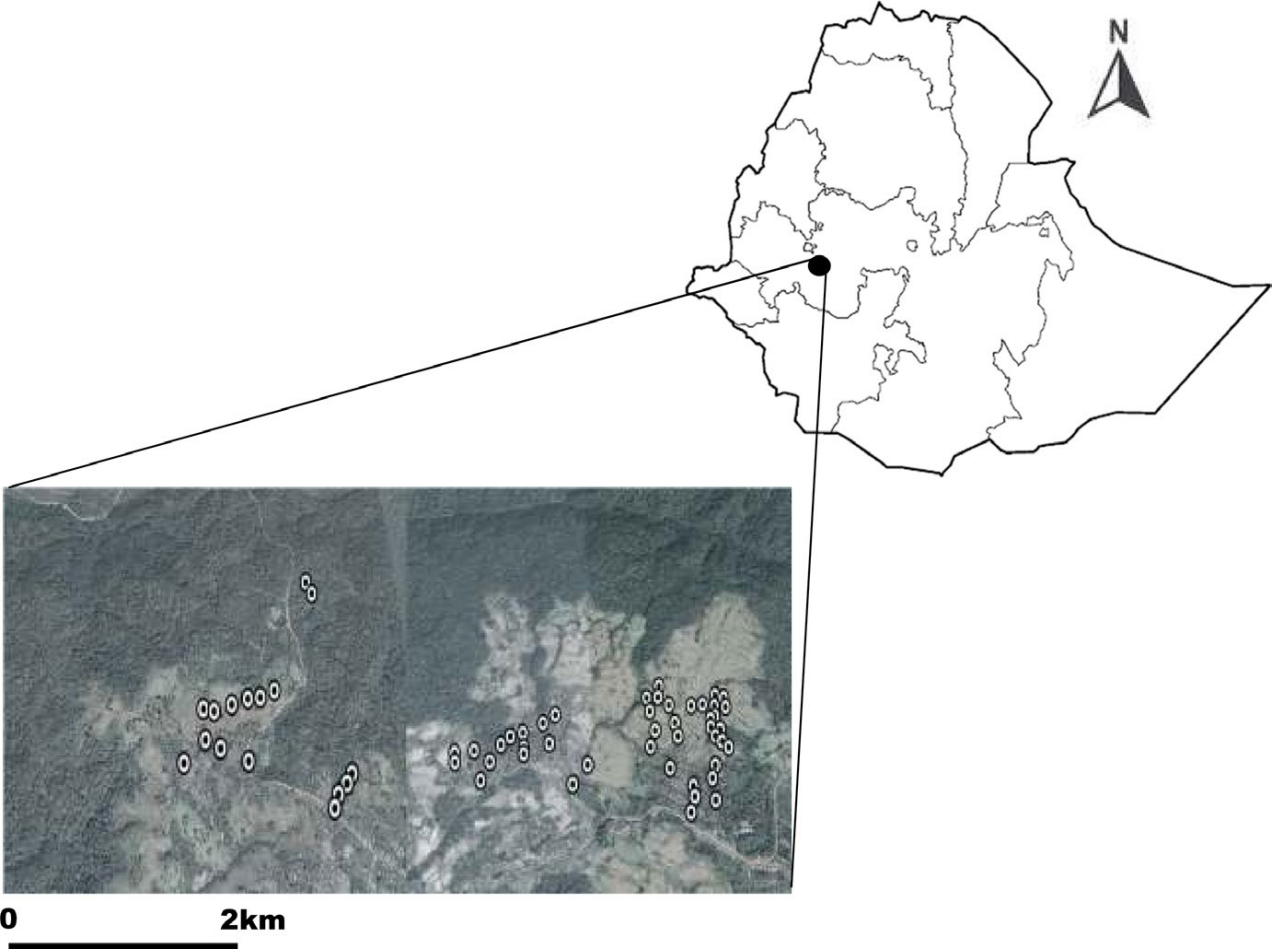
The map of the study area in relation to the map of Ethiopia produced from satellite image in Google Earth (Image©2020 CNES/Airbus), white dots are the location of the households used for interview, dark areas are forests, and white gray fields are agricultural areas in the landscape

The Yayu Coffee Forest Biosphere Reserve is located in Illubabor Zone of Oromia National Regional State, southwest Ethiopia between the geographical coordinates of 8°10ˈ0″N–8°20ˈ0″ N latitudes and 35°40ˈ0″ E‐36°0ˈ0″ E longitudes (Figure 1). The elevational range of the study area is between 1,140 and 2562 m a.s.l. (Gole et al., 2008). Yayu Coffee Forest belongs to the moist evergreen afromontane vegetation type (Friis et al., 2010) and was designated as biosphere reserve in 2010 by UNESCO (Gole et al., 2009). The total area of the Yayu Coffee Forest biosphere reserve is 16.7 Km^2^ (Fekensa et al., 2016). The major dominant woody species of the area include *Albizia* spp., *Cordia Africana, Pouteria adolfi‐friedericii*, *Ficus* spp., and *Polyscias fulva* (Senbeta, 2006) and the common wild large mammals found in the study area are lions, buffalos, bush pigs, warthog, forest giant hog, porcupines, olive baboons, and vervet monkeys (Lemessa et al., 2013; Quirin, 2005).

The area gets the mean annual precipitation of 2,100 mm that ranges between 1,400 and 3,000 mm with a unimodal pattern (Friis et al., 2010; The area has hot and humid climate and the mean annual temperature of 20°C where the minimum and maximum ranges between 12°C and 29°C Gole et al., 2009).

The local people largely depend on agriculture for their livelihoods and a mixed farming system is exercised in the area where production comprises coffee and cereal crops, animal husbandry, beekeeping, and spices. The major cereal crops include maize, sorghum, *teff*, wheat, burley, and millet, while coffee is the major cash crop accounting over 60% of the households' annual income (Gole, 2003; Fekensa et al., 2016).

### 2.2 Data collection

The first 1 km long transect was laid out along the edges of Yayu Coffee Forest. In the next, 29 transects (each 1 km long) were arranged with 200 m interval in parallel to the first transect and to each other in the agricultural landscape. These transects were categorized into three locations: ten transects close to forest (<0.5 km), ten intermediate (0.5–1 km), and ten transects far from forest (>1 km) in similar method used by Lemessa et al. (2013). By walking along each transect in zigzag way turning right and left, the households were randomly selected for questionnaire survey. Accordingly, from each three locations from forest edges (i.e., 48 households close to, 38 intermediate and 38 far away from forest households) or 2–6 households from each thirty transects and in total 124 households were interviewed using semistructured questionnaire. This household level interview was executed during the crop growing and maturity seasons from 1 February 2019 to 30 July 2019.

The major data collected were the type of crop raiders and the frequency of occurrences per year, crop species liable to be raided, extent of crop damage and how the households perceive the about the Yayu Coffee Forest. The scientific names of the large mammals were identified using field guide of large mammals of Africa after their local or common names in *Afaan Oromo* were collected during the questioner survey (Stuart, 2006). Moreover, after the maize crop was identified as the most liable to crop raiding, one maize field of each of 22, 21, and 21 households used for questionnaire survey was, respectively, selected from the three locations close to forest, intermediate, and far away from forest edge at random to estimate the extent of crop raiding. Correspondingly, four permanent plots (size: 4 × 4 m each) were randomly established in each of the 64 maize sown fields of the households (0.5 ha each on average) in March 2019 and at the maturity stage the number of damaged stems due to crop raiding were recorded in July, 2019. Moreover, the amount of annual yield loss estimated by respondents was compared with the yield loss we computed based on the secondary data of the average annual yield of maize from Yayu District Agricultural Development Office of the study area (YDADO, 2019). Here, the percent of annual yield loss due to crop raiding was calculated in percent from the difference between the amount of annual maize yield from the field survey (actual yield of maize) and average annual yield of maize obtained from YDADO.

### 2.3 Data analyses

The perception of the households on forests in relation to the impact of crop raiding at the three locations from forest edges (i.e., close to forest, intermediate, and far away from forest edges) was tested using Pearson's Chi‐square test. The number of maize stems attacked by crop raiders was used as a proxy indicator for the amount of damage by crop raiders. The number damaged maize stems were square‐root transformed to meet the assumption of homoscedasticity of the variance. To test for the variation in the amount of damage among the three locations (close to, intermediate, and far from forest edges) one‐way ANOVA was run and after the significant variation was found among the three locations, a multiple comparison of the means was made using HSD Tukey test with bonferroni *p*‐value adjustment. All data were analyzed using R‐statistical program version 4.0.2 (R Core Team, 2020).

## 3 RESULTS

### 3.1 Crop raiders and crops most raided in relation to distances from forest edges

Four wild large mammal pests, such as olive baboon (*Papio anubis*), vervet monkey (*Chlorocebus aethiops*), bush pig (*Potamochoerus larvatus*), and crested porcupine (*Hystrix cristata*) were identified by all of the respondents as the worst crop raiders in the area. According to the respondents, the crops that are most raided by these and other wild large animals are maize, sorghum, potato, mango, avocado, and *teff*. In this connection, the respondents close to forests (67%), at intermediate distance (34%), and the respondents at far away from forests (42%) mentioned that from among the crops identified above, maize is reported as the most liable crop to be raided by wild large mammals in the area (Table 1).

**TABLE 1:** Frequency of farmers' responses in percent on types of crops often raided in relation to distances from forests

Locations	Frequency of farmers' responses on types of crops raided (%)					
	Maize	Sorghum	Potato	Mango	Avocado	Teff
Close to forest	66.7	4.2	6.2	4.2	14.6	4.2
Intermediate	34.2	21	7.9	13.2	13.2	10.5
Far from forest	42.1	21	7.9	7.9	0	7.9

### 3.2 The pattern of crop raiding with distances to forest edges

**FIGURE 2:**
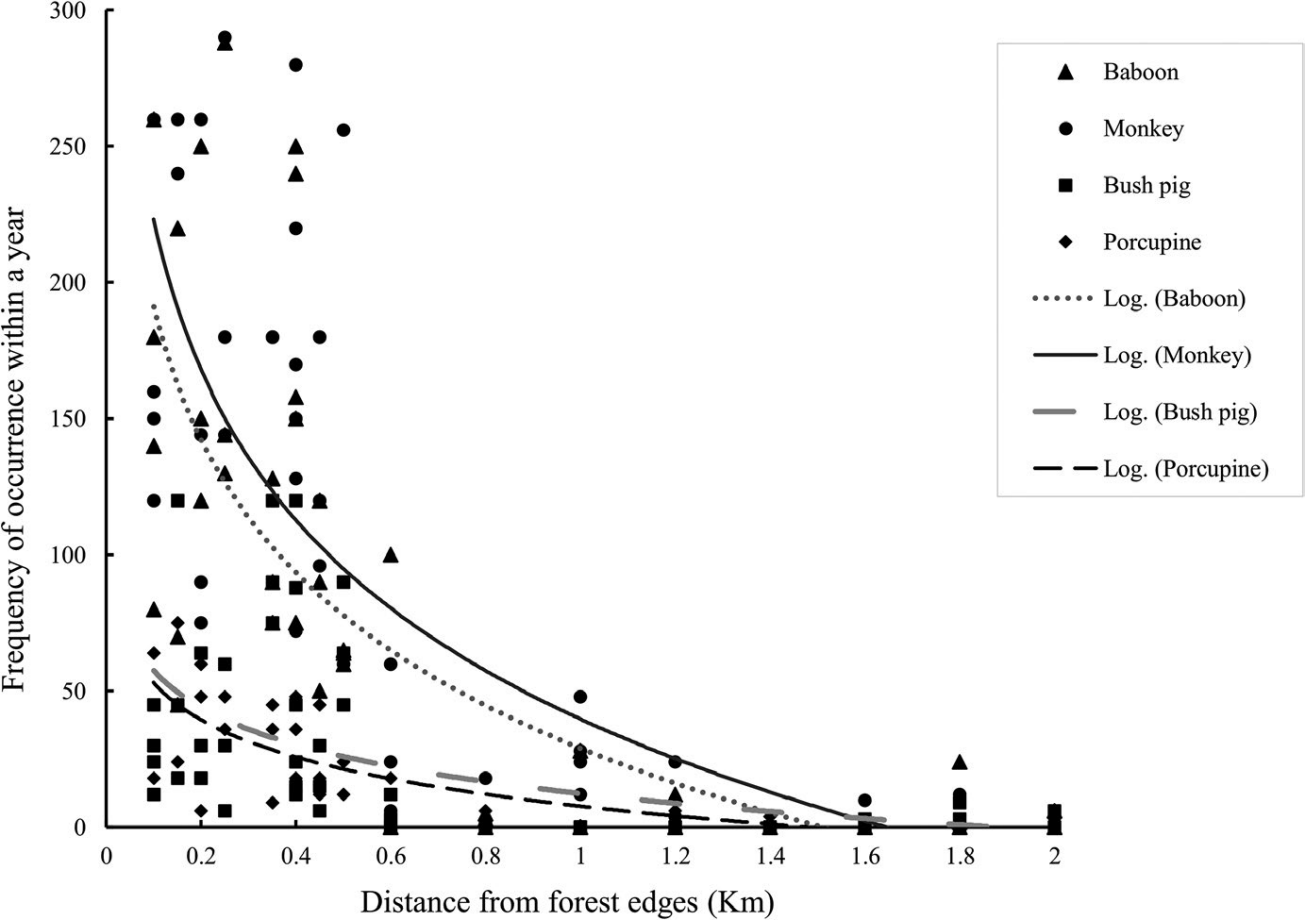
The line graph showing the frequency occurrence of the four top wild large mammal crop raiders (olive baboon, vervet monkey, bush pigs, and crested porcupine) in agricultural fields

**FIGURE 3:**
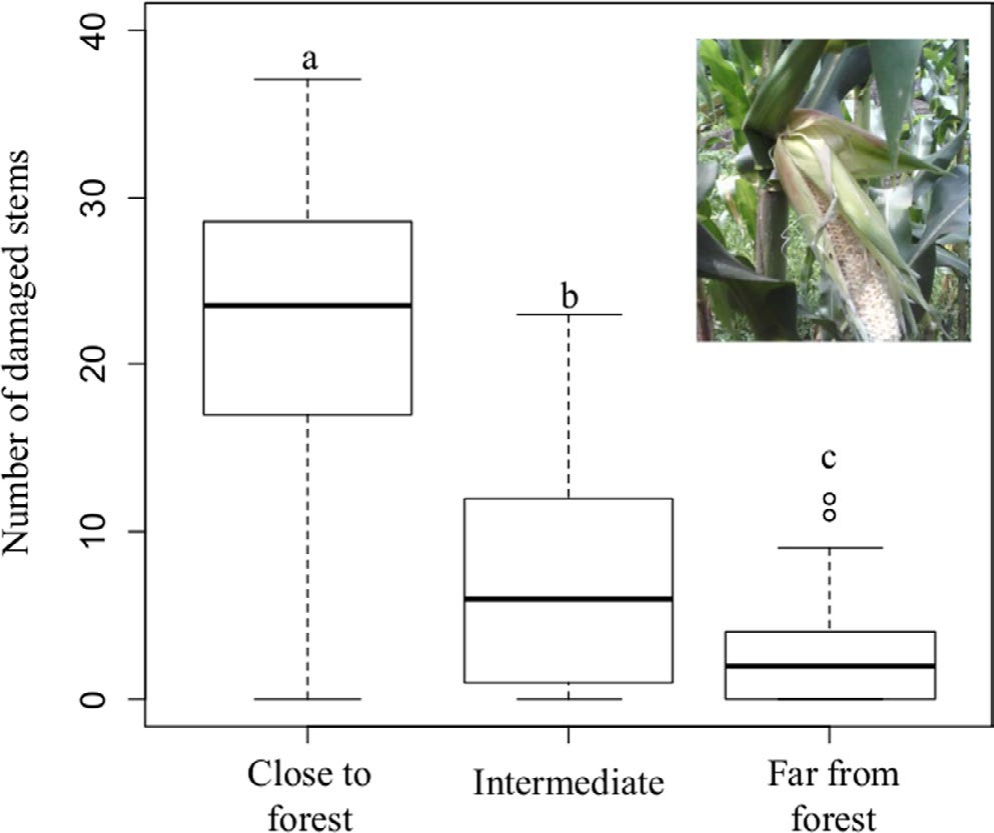
The boxplot showing the number of stems (≈number cobs) damaged within plots (4 × 4 m) by crop raiders in relation to different distances from forest edges. Different small letters on the boxplot shows significant differences (*p* < .001)

The frequency of occurrence of crop raiders in agricultural field varies spatially and decreases with increasing distance from the forest edge (Figure 2). The severity of crop raiding is dependent on the different locations from the forest edges (*χ* ^2^ = 8.04, *df* = 2, *p* = .018). The households close to the forests face severe crop raiding impact than those living far away from Yayu Coffee Biosphere Reserve. Here, the respondents living close to forests (89.6%) and at intermediate distances from forests (28.9%) estimated that the annual maize yield loss due to crop raiding ranges between 20% and 40%, while 100% of the respondents dwelling at far away from forests indicated that the annual maize is <5% (Table 2). These results of the annual yield loss per ha estimated by respondents are also close to the amount of yield loss computed from field survey and based on the expected annual yield of maize gathered from the Yayu district agricultural development office that the average annual maize yield loss per ha was 26.4% (881 kg/ha) close to forest, 11% (366 kg/ha) at intermediate and 4.1% (137 kg) of the total expected yield at a sites far away from forest edges. The result of one‐way ANOVA showed that the amount of damage in maize fields was higher close to forest when compared with the level of damages located either at intermediate or far from forest edges (*p* < .001, Figure 3).

**TABLE 2:** The annual maize yield loss per hectare estimated by respondents (%) in relation to the location from forest edges

Yield loss (%/ha)	Distance from the forest edges					
	Close (<0.5 km)		Intermediate (0.5–1 km)		Far away (>1 km)	
	Frequency		Frequency		Frequency	
	(*N* = 48)	%	(*N* = 38)	%	(*N* = 38)	%
20–40	43	89.6	11	28.9	0	0
10–20	5	10.4	7	18.4	0	0
5–10	0	0	14	36.8	0	0
<5	0	0	6	15.8	38	100

The perception of respondents on forests vary among the different locations from forest edges that respondents living close to forests (81%), at intermediate distance from forests (65.8%), and respondents situated at far away from forests (52.6%) of, explained that forest is home for wild large mammals and hence is a threat to our survival (Table 3). However, how the respondents perceive the trend of crop damage by wild large mammals vary among respondents in that 91.1% (113) of the respondents stated that the extent of damage is increasing while 8.9% (11) of the respondents assume that it is not possible to predict.

**TABLE 3:** The perception of the respondents on forests in relation to different distances from forest edges

Location	Number of respondents (*N*)	Forest is threat to our life		Forest is useful for our life	
		*N*	%	*N*	%
Close to forest	48	39	81.25	9	18.75
Intermediate	38	25	65.8	13	34.21
Far from forest	38	20	52.6	18	47.37
Total	124	84		40	

## 4 DISCUSSION

Understanding how crop raiding varies across spatial scales from forest edges is critically essential in subsistence farming system to frame harmonized development‐conservation schemes. We show that the pattern of crop raiding and perceptions toward forest vary with distances from forest edges. The frequencies of crop raiding by wild large mammals surrounding Yayu Coffee Biosphere Reserve in southwest Ethiopia decrease from forest edges and flatten after 2 km. Similarly, the level of crop damage is higher at sites close to forests and at intermediate distances from forests when compared with the sites at far away from forests. Our results corroborate several previous findings from southwest Ethiopia that the frequency of occurrence of wild mammal crop raiders and the corresponding extent of crop damage decrease with increasing distances from forest edges into agricultural fields (Ango et al., 2017; Geleta et al., 2019; Lemessa et al., 2013; Leta et al., 2016). However, there is a variation in the magnitude of distances that crop raiders move out from their natural habitats into agricultural fields including in homegardens for raiding different kinds of crops. Our study indicated that not only the frequency of occurrence and extent of crop damage close to the forest is higher for diurnal crop raiders such as olive baboons, and vervet monkeys, but also their foraging range is farther away from forest edges when compared with those of nocturnal crop raiders including crested porcupines and bush pigs (Figure 2). Related to this, several studies have reported that diurnal wild large mammals have wider spatial raiding range and cause huge damage to crops and disrupt the livelihood of the farmers in Africa (Joseline, 2010; Kate, 2012; Datiko & Bekele, 2013). In this connection, our results contradict the earlier finding of Lemessa et al. (2013) that denoted that nocturnal mammal crop raiders move longer distances into agricultural fields for raiding than diurnal mammals for the reason that protecting crops during the night is weaker.

Interestingly, in line with our hypothesis, the severity of crop raiding and the level of crop damage are higher close to forests than at far away from forests. Here, 89.6% (48) of the farmers estimated the annual maize yield loss due to the crop raiders to be between 20% and 40%, per hectare close to forests and 28.9% (11) at intermediate locations and none of the respondents reported at far away from forests (Table 2). On the hand, 100% of the respondents at far away from forests asserted that the yield loss due to crop raiding is <5%/ha. Our attempt to counter check these farmers estimation with the annual yield loss from field survey and expected yield data obtained from Yayo district agricultural development office also affirms that the annual yield loss of maize due to crop raiders vary spatially as 26.4% (881 kg/ha) close to forest, 11% (366 kg/ha) at intermediate and 4.1% (137 kg) of the total expected yield at a sites far away from forest edges. Moreover, this farmers estimation is also pretty similar with the earlier finding of Ango et al. (2017) who measured the annual yield loss of maize per ha and reported to be 243 kg (34%) of the total expected annual yield per ha close to forests and 80 kg (11.5%) at away from forests in southwest Ethiopia. Moreover, the present estimation is also close to what was reported from Uganda that the seasonal amount of yield loss by wildlife reaches up to 50% (Wallace & Hill, 2007).

The spatial variation in the resultant impacts of the crop raiding is reflected on how farmers perceive forests. In view of this, about 81% of the respondents dwelling close to forest perceive that forests host wild animals that are threat to their life while 47.4% of the respondents located at far away from forests explained that forests are useful for their life (Table 3). Such heightened negative perception conceived by farmers living close to forest, in fact, emanates not only from the challenges that crop raiders are competing for their crops and livestock depredation, but also elevated by the anxiety that comes from guarding crops day and night, unable to send their children to schools, their minds are always restless and thereby their health is affected (Ango et al., 2017; Hariohay & Røskaft, 2015; Hill, 2000; Linkie et al., 2007). Consequently, negative perceptions are reflected in undermining the conservation of wildlife and forests and retaliator killing of wild animals and clearing of forests are the indicators of such reaction or developed behavior (Carter et al., 2014). Nevertheless, as related by Hill (2017), the negative interaction between humans and wild animals may depend on the several factors which could include the size of the habitats at the ecotones of agriculture‐natural landscapes, availability of food in the wild nature and the types of crops farmers grow at forest edges and food preferences of the wild animals.

Our results depicted that maize is the most raided crop at different distances from forest edges and according to our respondents the reason for this might be maize is sweet and more palatable compared to other crops. As regards this, the study conducted in Uganda stated that baboons prefer maize for raiding throughout the year when it is available in agricultural fields relative to other crops (Hill, 2000). Unequivocally, the extent of crop damage caused by wild large mammal crop raiders, in general, is higher close to forests as also denoted by Hill (2017) from the United Kingdom that crop damage is heightened within 200 m of the forest edges. However, regardless of the spatial variation, the wild large mammal crop raiders cause damage to crops by both eating and trampling at different growth stages. For example, baboons and porcupines raid crops at all growth stages from the seedlings to the maturity, monkeys, and bush pigs causes more damage during the flowering and maturity stages of crops (according to our respondents). In this regard, Hill (2017) related that primates' crop foraging activity depends on crop varieties and growth stages. For all, the adversity of crop raiding is more magnificent in regions where small scale subsistence farming system is largely practiced as in the case of Africa (Hill, 2004, 2005). Our study shows that the severity of crop raiding is highly pronounced in areas where agricultural fields found close to forests. Similarly, posed by strong negative interactions at these areas, the negative perception toward forests and wildlife is found to be more significant close to forests than at far away in agricultural fields. By sum, unless crop raiding is managed synergizing the socio‐ecological systems, the impacts may defy farmers' livelihoods and the negative attitude especially at crop fields‐wild nature ecotones could in turn lead to the loss of biodiversity and the associated ecosystem services.

## 5 CONCLUSION

Crop raiding triggers a human‐wildlife conflict mainly in the ecotone areas of human‐modified natural landscapes. Understanding the spatial pattern of crop raiding from forest edges is critically essential in subsistence farming system to frame harmonized development‐conservation schemes. The present study showed that the intensity of crop raiding and how farmers perceive forests vary with distances from forest edges. In sum, our results suggest that strategies need to be sought in order to minimize the socio‐ecological impacts of crop raiders mainly in locations close to forest edges.

## CONFLICT OF INTEREST

None declared.

## AUTHOR CONTRIBUTION

**Alemayehu Mamo:** Data curation (lead); Project administration (lead); Writing‐original draft (equal). **Debissa Lemessa:** Formal analysis (lead); Methodology (lead); Validation (lead). **Obsu Hirko Diriba:** Funding acquisition (lead); Supervision (equal); Writing – review and editing (equal). **Debela Hunde:** Supervision (equal); Validation (equal); Writing – review and editing (equal).

## ETHICAL APPROVAL

This study is based on the questionnaire survey and observation or assessment of the crops damaged by large mammal pests and did not involve human subjects, experimentation with animals or collection of specimens. We all authors abide by the Ethical standards stated in the Ecology and Evolution journal Guidelines for Authors.

## Data Availability

All data used in the manuscript were deposited in Dryad repository with https://doi.org/10.5061/dryad.tht76hdz3
